# Rapid determination of nutrient composition and mineral element content of common vetch (*Vicia sativa* L.) using near-infrared spectroscopy

**DOI:** 10.5713/ab.24.0872

**Published:** 2025-04-11

**Authors:** Tao Wang, Li Wang, Tao Guo, Yanli Shi, Baocang Liu, Fei Li

**Affiliations:** 1 State Key Laboratory of Grassland Agro-ecosystems, Key Laboratory of Grassland Livestock Industry Innovation, Ministry of Agriculture and Rural Affairs, Engineering Research Center of Grassland Industry, Ministry of Education, College of Pastoral Agriculture Science and Technology, Lanzhou University, Lanzhou, China; 2 Xinjiang Taikun Group Chang Feed Co., Ltd., Changji Hui Autonomous Prefecture, China

**Keywords:** Common Vetch, Modified Partial Least Squares Method, Near Infrared Spectroscopy, Nutritional Quality, Prediction Accuracy

## Abstract

**Objective:**

This study aims to establish an accurate and reliable near-infrared spectroscopy to enable rapid, efficient, and non-destructive evaluation of the nutritional quality of common vetches across different regions and varieties.

**Methods:**

A total of 190 samples from various regions and varieties were selected for this study, which were divided into a calibration set (4:1 ratio) and validation set. The original spectrum of the calibration set is subjected to 10 different pretreatment techniques in combination with first and second derivatives, while the prediction model was established by combining the measured value of common vetch with the modified partial least squares method.

**Results:**

The results indicate that the calibration root-mean-square error and cross-validation root-mean-square error values range from 0.01 to 1.25 and 0.01 to 1.37, respectively. The determination coefficients of cross-validation (R^2^CV) for phosphorus (P), potassium (K), magnesium (Mg), and iron (Fe) are relatively low at 0.82, 0.86, 0.82, and 0.74, respectively; however, all other indicators have R^2^CV values above 0.90. The predicted root means square errors (RMSEP) for common vetch indexes range from 0.01 to 1.87, with RMSEP values higher than 1.0 observed for crude protein, neutral detergent fiber, acid detergent fiber, and ash indices, whereas RMSEP values lower than or equal to 1.0 were obtained for other indicators. The measured coefficient of determination (R^2^_p_) demonstrates that the R^2^_p_ values for each nutrient element and mineral element vary from 0.70 to 0.96. The residual prediction deviation (RPD) values for Mg exhibit relatively low levels, while the RPD values for other indicators exceed 2.0.

**Conclusion:**

These findings suggest that this study provides a viable approach to evaluate the nutritional composition and mineral element content of different varieties and regions of common vetch.

## INTRODUCTION

Common vetch, also known as the big nest vegetable or wilderness pea, is an annual or perennial species in the legume family and belongs to the genus Vicia [1]. Common vetch is noted for its resilience to cold, drought, and infertile soils. In northwest China, Common vetch is widely integrated into wheat fields through intercropping and multiple cropping systems to enhance protein content and pasture yield [2]. As global living standards improve, the demand for ruminant meat rises, driving the need for high-quality forage. Recent domestic and international research has demonstrated that feeding common vetch or blending it with Gramineae grasses can significantly improve the palatability and digestibility of feed, and enhance ruminants’ performance through adjusting rumen microbial communities [3]. As a result, common vetch has become a crucial source of premium forage in animal husbandry [4]. To address feed ingredient shortages, breeders and scientists have focused on developing common vetch varieties with enhanced stress tolerance, high yields, disease resistance, and nutritional richness over recent decades [5,6]. The findings of this study have facilitated the development of a diverse array of common vetch varieties in the market, which offers producers an expanded selection and allows for optimized feed production in accordance with regional demands [7].

However, the determination of nutrient content in common vetch still relies on traditional wet chemical analysis, which not only consumes a significant amount of time and resources but also imposes specific requirements on experimental equipment and operators [8]. The absence of a rapid, convenient, and cost-effective method for quality evaluation of common vetch presents challenges for producers in accurately selecting the desired varieties promptly. Therefore, there is an urgent need to develop an efficient, accurate, simple, and affordable method to evaluate the nutritional value of common vetch. This endeavor holds great significance in promoting the rapid development of the common vetch industry.

The near-infrared reflectance spectroscopy (NIRS) is not only characterized by its simplicity, speed, efficiency, accuracy, and cost-effectiveness but also eliminates the need for complex pre-treatment and chemical analysis processes. Additionally, it enables simultaneous analysis of multiple chemical indices [9]. In recent years, research has demonstrated the effective utilization of near-infrared spectroscopy combined with chemometrics for the comprehensive analysis of plant chemical composition and high-throughput phenotype [10]. However, previous studies primarily focused on selecting samples from the same variety of plants or feeds, resulting in minimal variations in various chemical indexes among these samples. This could potentially hinder the accurate prediction of nutritional indexes for different varieties within the same variety by the established model [11]. Therefore, there is still limited available literature on the utilization of NIRS models for predicting nutrient composition and mineral element content in different varieties and regions of common vetch.

In this context, the objective of this study was to develop a rapid, efficient, and non-destructive quantitative analysis model for nutrients and mineral elements in common vetch from diverse regions and varieties by integrating near-infrared spectroscopy with chemical composition analysis. We hypothesized that through extensive sampling of common vetches from various regions and varieties, a rapid, accurate, and reliable NIRS model could be established to effectively predict the nutritional quality of common vetches. This model is expected to accurately predict the nutritional quality of common vetch, thereby providing a solid theoretical foundation and advanced technical support for the selection of high-quality varieties. This will significantly advance the development of the common vetch-based forage industry.

## MATERIALS AND METHODS

### Sample selection and preparation

To construct an optimal near-infrared NIR model, a meticulously curated dataset of 150 samples was extracted from diverse common vetch varieties cultivated at the Yuzhong Campus of Lanzhou University (Lanzhou, China). This collection included the varieties Lanjian 1, Lanjian 2, Lanjian 3, Lanjian 6, and Lanjian 7, among others. Furthermore, to ensure the representativeness of the samples, an additional 40 samples were collected from various regions of Qinghai Province, which expanded the sample collection to a total of 190, representing a broad spectrum of ecological niches and growth conditions. This extensive sampling coverage guarantees the robustness and versatility of the NIR model.

During the mature period and heading stages of selected common vetch, samples are manually collected by cutting the plants at a height of 3 to 5 cm to ensure representative sampling. Subsequently, the stem samples are promptly transported to the laboratory for analysis and processing. In the laboratory, the stem samples are initially placed in a 65°C oven to remove excess moisture and maintain their stability. Subsequently, the common vetch stem samples were trimmed into fragments measuring 2 to 3 cm in length and pulverized using a grinder (CM100; Crinoer Technology, Beijing, China). Furthermore, as an essential step in the grinding process, it is crucial that the samples are screened with a 1 mm screen to further refine them according to specific particle size requirements. After completing these procedures, it is necessary to thoroughly blend the mixture before storing it in an air-tight bag while ensuring protection from light exposure and maintaining optimal dryness and environmental conditions for quality stability.

### Determination of nutritional and elemental composition of samples

These samples were analyzed to dry matter (DM; method 930.15), crude protein (CP; method 976.05), ether extract (EE; method 2003.05), ash (method 942.05), neutral detergent-insoluble fiber (NDF; method 2002.04), and acid detergent-insoluble fiber (ADF), as described in AOAC [12]. The content of organic matter (OM) is calculated by subtracting the ash content from the DM value. The samples were subjected to two repeated measurements, and the results were presented as an average value. Furthermore, all results were standardized on a DM basis.

According to the Chinese National recommended standard (GB/T 20902-2014), the mineral element content was determined using the following procedure: Firstly, weigh 0.3 g (accurate to 0.0001 g) of the common vetch sample that has been passed through a 1mm sieve and uniformly mixed, and place it into the microwave digestion vessel. Subsequently, 8.0 mL of high-grade pure concentrated nitric acid and 1.0 mL of high-grade pure hydrogen peroxide were added, allowing to stand for 30 min to ensure thorough reaction. The digestion process was carried out using a microwave digester (JUPITER-B; Shanghai Xinyi Microwave Chemical Technology, Shanghai, China). The digestion procedure consisted of two stages: initially maintaining the temperature at 150°C for 10 min, followed by raising it to 180°C for an additional 15 min until complete dissolution of the sample was achieved. Subsequently, the digested sample was transferred to an acid separator (TK12; Shanghai Xinyi Microwave Chemical Technology) and subjected to deacidify treatment at 160°C. When the liquid volume in the microwave digestion tank reduced to approximately 1 mL, the sample was removed and allowed to cool down to room temperature. The resulting digestive solution was then transferred into a 50 mL volumetric bottle, set with deionized water to constant volume, and filtered through a membrane filter with a pore size of 0.45 μm. Subsequently, in accordance with the Chinese national recommended standard (GB/T 30902-2014), calibration was performed using the inorganic element mixed solution reference material (GBW (E) 081531, China National Institute of Metrology, Beijing, China) and the phosphorus element reference material (GSB04-1741-2004 (b), China National Institute of Metrology, Beijing, China), both provided by the Chinese Academy of Metrology Sciences. The determination was carried out using an Inductively Coupled Plasma Optical Emission Spectrometer (ICP-OES 725; Agilent Technologies, Santa Clara, CA, USA), with a multi-color temperature set at 35°C. The plasma gas flow rate, nebulizer gas flow rate, and auxiliary gas flow rate were adjusted to 12, 1 and 1 L/min respectively. The spectral selection, standard curve, and correlation coefficient for each element are detailed in Table 1. Each sample was measured twice in parallel, and the average value was taken as the final content of the sample elements. Blank controls were also established to ensure accuracy of the results.

### Near-infrared reflectance spectroscopy spectral data acquisition and pretreatments

A multifunctional FOSS NIR-Systems DS2500 (FOSS Electric A/S; FOSS, Hillerod, Denmark) was used to scan the ground samples of common vetch. Spectral images were collected within the spectral range of 850 to 2,500 nm and with a spectral resolution of 0.5 nm. Prior to sample scanning, it is necessary to power on and preheat the instrument, followed by inspecting it using a standard sample cup once the performance test meets the qualification criteria to ensure normal operation. Subsequently, thorough cleaning of both the sample cup and test window is essential before filling the sample cup approximately two-thirds full for scanning initiation. Each sample should undergo two scans, with their average spectrum being considered as the final NIR scan spectrum for common vetch.

The WinISI IV (version 4.6.11; developed by Infrasoft International, Silver Spring, MD, USA) software was utilized in this study to establish, validate, and evaluate the prediction model for nutrient composition and mineral elements of common vetch. Initially, principal component analysis (PCA) was conducted on the average spectra of all samples to eliminate any abnormal spectra. Abnormal data were identified by applying a Global distance threshold (Global Hood≥3) and a T value criterion (T>2.5), resulting in the screening of spectral values. The WinISI IV (version 4.6.11; developed by Infrasoft International) software applies two derivative processing techniques and ten de-scattering methods to mitigate the influence of external factors on the spectrum, which methods include no operation at all (none), quadratic derivative range compensation (scale and quadratic), standard normal variable transformation combined with scattering correction (SNV and detrend), linear derivative range compensation (scale and linear), standard normal variable change only (SNV only), weighted MSC with scale and offset adjustment, detrend only operation, inverse discrete correction MSC operation, and standard multivariate discrete corrective treatment using MSC [13,14]. Mathematical treatments are hereafter referred to using numerals, such as 1, 4, 4, 1, in which numbers respectively represent the quantity of derivatives to be calculated, the discrepancy involved in derivative computation, the number of data points utilized for moving average or smoothing operations, and finally the number of points employed in secondary smoothing. In total thirty pretreatment techniques were utilized [15].

### Near-infrared reflectance spectroscopy modeling and performance evaluation

In this investigation, we randomly partitioned the preprocessed spectra into a calibration set (n = 152) and a validation set (n = 38) at a 4:1 ratio. The optimal spectral processing method was chosen by modified partial least squares method (MPLS), and the spectral absorption values were integrated with measured analytical values from the laboratory for regression analysis in constructing the prediction model. The assessment of model performance relies on the following metrics: calibration root means square error (RMSEC), cross-validation, predicted root mean square error (RMSECV and RMSEP), coefficient of determination (R^2^_C_) for calibration, coefficient of determination (R^2^_CV_) for cross-validation, coefficient of determination (R^2^_p_) for prediction, and residual prediction deviation (RPD) [16]. Among, a higher R^2^_CV_ value, along with lower RMSEC and RMSECV values, indicates a more effective model. The closer the predicted coefficient of determination (R^2^_p_) is to 1.0, the higher the accuracy of the model; when 0<R^2^_p_≤ 0.25, it indicates that the established predictive model is not feasible [17]. During the validation stage, the model’s accuracy was assessed using RPD. An RPD>2.5 indicates exceptional performance and suitability for quality evaluation. If the 2.0≤RPD≤2.5, it suggests that the model is suitable for preliminary analysis of forage quality. If the RPD<2.0 indicates inadequate predictive performance, rendering the model unsuitable for practical use [18].


(1) $$\text{RPD}=\frac{\text{SD}}{\text{REMSEP}}$$

In this equation, SD denotes the standard deviation of the reference values within the validation set.

### Statistical analysis

All experimental data were analyzed using IBM SPSS Statistics 26.0 (version 26.0; IBM, Armonk, NY, USA). The data statistical model was constructed using common vetch samples as a fixed variable and each replicated as a random variable. The experimental unit was defined as the number of samples in both the calibration and validation sets. The nutritional composition and mineral element content of the common vetch sample were analyzed with SPSS 26.0 (version 26.0; IBM, Armonk, NY, USA), including max, min, mean and standard deviation (SD). The spectral data preprocessing and prediction model for nutrients and mineral elements of common vetch will be established with WinISI IV (version 4.6.11; developed by Infrasoft International) software. Finally, the corresponding diagrams are generated by Origin 2019 (Origin Lab, Northampton, MA, USA) software along with SIMCA (Sartorius Data Analytics, Malmö, Sweden) software.

## RESULTS

### Main compositions of common vetch samples

The nutrient composition and mineral element contents of common vetch are presented in Table 2. The coefficients of variation (CV) for CP, ash, iron (Fe), potassium (K), and phosphorus (P) were relatively high, at 29.70%, 28.53%, 55.93%, 33.94% and 28.00%, respectively. In contrast, the variability of DM, NDF, ADF, EE, OM, calcium (Ca) and magnesium (Mg) was relatively low with CV at 0.73%, 13.34%, 14.78%, 18.75%, 26.55% and 17.14 % respectively. Additionally, the SD for all indicators ranged from 0.06 to 5.37.

### Spectral analysis

**Figure 1:**
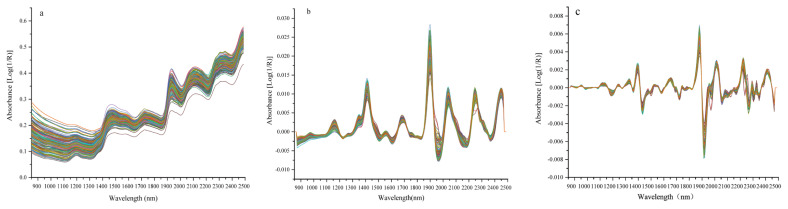
Original and preprocessed average NIR absorption spectra of common vetch samples in the range of 900 to 2,500 nm. (a) The original spectrum of the common vetch samples; (b) Spectrum after first derivative treatment of common vetch samples; (c) Spectrum after second derivative treatment of common vetch samples. NIR, near-infrared.

**Figure 2:**
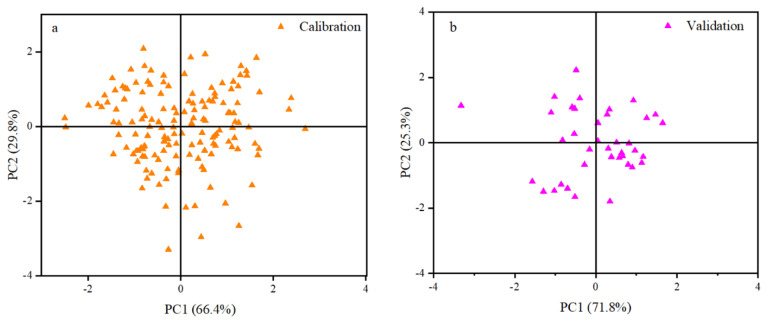
PCA score of common vetch samples in calibration and validation set. (a) Calibration set of PCA scores for the samples of common vetch; (b) Validation set of PCA scores for the sample of common vetch. PCA, principal component analysis.

The mean near-infrared absorption spectra of the common vetch sample within the wavelength range of 850 to 2,500 nm are presented in Figure 1. As depicted in Figure 1A, the common vetch sample exhibits five distinct absorption peaks across the entire spectral range, specifically located at approximately 1,450, 1,740, 1,910, 2,120, and 2,350 nm respectively. Based on the findings illustrated in Figures 1B, 1C, it is evident that derivative processing significantly enhances the characteristic spectral peak. Furthermore, a comprehensive investigation of the spectral data of common vetch samples was conducted using PCA. The distribution of the primary components in NIR spectrum within both the calibration set and validation set is depicted in Figures 2A, 2B, respectively. These results obtained from PCA demonstrate the homogeneity and well-mixed nature of the principal components, accounting for more than 96 % of the spectral variance. Notably, Figure 2A reveals greater variations in the spectrum of the calibration set, emphasizing the representativeness of selected samples and facilitating optimization of NIR spectral modeling process.

### Development of calibration models for common vetch

In subsequent NIRS modeling, the calibration set, and validation set were determined based on the MPLS score. One sample was randomly selected from each five to be included in the validation set, while the remaining samples were used to construct the calibration set. As illustrated in Figure 2, the reference values of the validation set and calibration set samples exhibit comparable distributions and ranges, indicating that the validation set, and calibration sets are suitable for the establishment and verification of the NIRS equation. Consequently, this study employed a combination of MPLS technology, mathematical processing techniques, and scatter correction methods to establish each respective of 30 equations that correlated spectral data with nutrient and element content of common vetch. The optimal equation should be identified based on the value of R^2^_CV_. The specific results are as follows: for DM, the optimal combination of spectral processing and derivative processing is Scale and Linear (1, 4, 4, 1), with an R^2^_CV_ of 0.90; for CP, the optimal combination is Weighted MSC (1, 4, 4, 1), with an R^2^_CV_ of 0.96. The optimal combination for NDF is Inverse MSC (0, 0, 1, 1), with an R^2^_CV_ of 0.93. For ADF, the optimal combination is SNV and Detrend (1, 4, 4, 1), with an R^2^_CV_ of 0.94. For EE, the optimal combination is SNV and Detrend (1, 4, 4, 1), with an R^2^_CV_ of 0.90. The optimal combination for OM is Weighted MSC (1, 4, 4, 1), with an R^2^_CV_ of 0.97. The optimal combination of Ca is Detrend only (1, 4, 4, 1), with an R^2^_CV_ of 0.92. Similarly, the optimal combination of P is Detrend only (1, 4, 4, 1), with an R^2^_cv_ of 0.82. The optimal combination of K is also Detrend only (1, 4, 4, 1), with an R^2^_CV_ of 0.86. The optimal combination of Mg is Weighted MSC (0, 0, 1, 1), with an R^2^_CV_ of 0.82. The optimal combination of Fe is Scale and Quadratic (1, 4, 4, 1), with an R^2^_CV_ of 0.74. Furthermore, the quantitative correction model, which was established using near-infrared spectroscopy, employs a series of evaluation parameters, including REMSEC, R^2^_C_, REMSECV, and R^2^_CV_. Of these, R^2^_C_ and R^2^_CV_ are approximately equal to 1, and RMSEC is approximately equal to 0, indicating that the constructed model is highly robust. A lower RMSECV value indicates a higher level of accuracy in the model’s predictive capabilities. The results of these evaluation parameters are presented in detail in Table 3. The ranges of each indicator of REMSEC and REMSECV are 0.01–1.25 and 0.01–1.37, respectively. Notably, the R^2^_C_ values for P, Mg, and Fe are relatively low, at 0.89, 0.88, and 0.74, respectively, while the R^2^_C_ values of the remaining indicators are all greater than 0.90. The values of P, K, Mg, and Fe in R^2^_CV_ are relatively low at 0.82, 0.86, 0.82, and 0.74, respectively, while the R^2^_CV_ of the other indicators are all above 0.90.

### Near-infrared reflectance spectroscopy prediction capability

**Figure 3:**
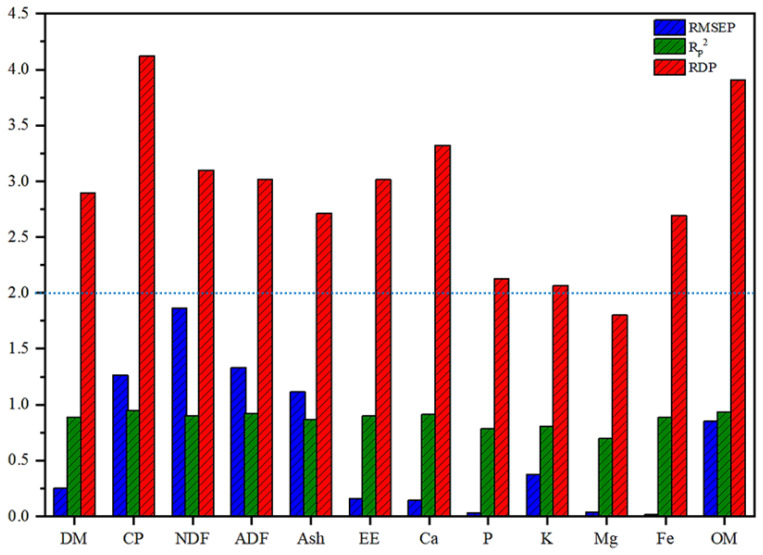
Predictive performance of MPLS Model for the nutrient and mineral element content in common vetch. RMSEP, root mean square error of prediction; R_p_^2^, correlation coefficient of validation set; RPD, the ratio of prediction to deviation; DM, dry matter; CP, crude protein; NDF, neutral detergent fiber; ADF, acid detergent fiber; EE, ether extract; OM, organic matter.

**Figure 4:**
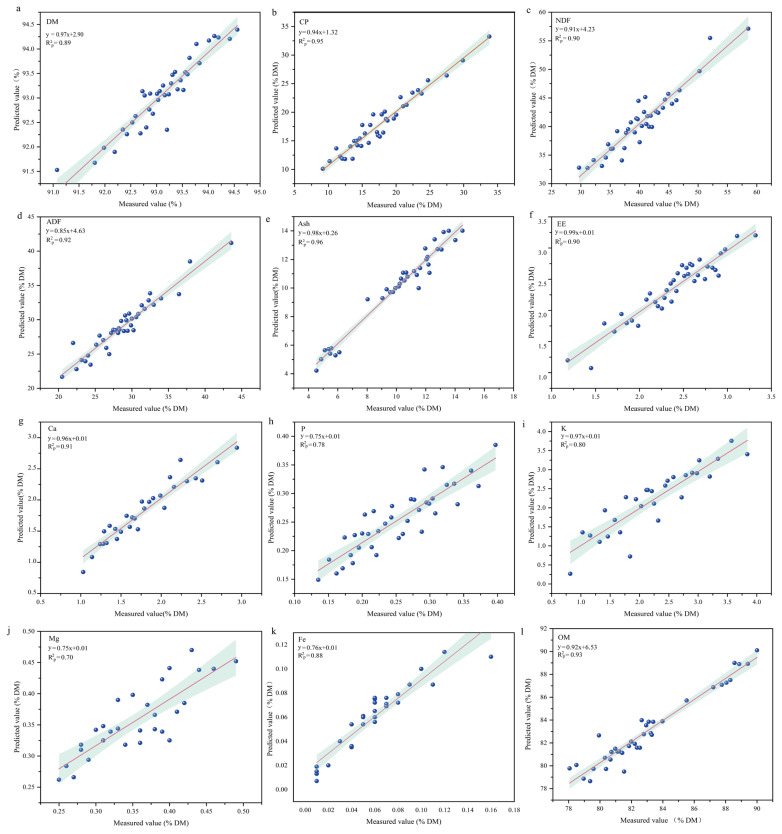
The correlation between the predicted values and measured values of nutritional composition and mineral element content in the optimal model of common vetch was investigated. R^2^_p_, correlation coefficient of validation set; DM, dry matter; CP, crude protein; NDF, neutral detergent fiber; ADF, acid detergent fiber; EE, ether extract; OM, organic matter.

The external validation set evaluation of the best model demonstrates its predictive ability, with relevant parameter values presented in Figure 3. RMSEP values for all indices ranged from 0.01 to 1.87, with CP, NDF, ADF and ash exhibiting higher RMSEP values above 1.0 while other indices displayed lower RMSEP values below 1.0. Correlation coefficients (R^2^_p_) between actual measured and predicted values are shown in Figure 4; the R^2^_p_ values for K and Mg were 0.78, 0.80, and 0.70, respectively, indicating relatively modest prediction accuracy. Conversely, the R^2^_p_values for other nutrients and mineral elements ranged from 0.88 to 0.96. RPD values for P, K and Mg were all less than or equal to 2.5 whereas RPD value of other indicators was greater than or equal to 2.5 (Figure 3).

## DISCUSSION

Currently, the breeding and genetics of common vetch have primarily focused on the adaptability and yield of different varieties in various regions, while neglecting the significance of nutritional quality and mineral elements [19]. Research has indicated that common vetches are extensively cultivated worldwide and possess abundant nutrients and mineral elements [20]. The rapid screening of the relationship between adaptation and nutritional quality in various regions is increasingly imperative. Among the promising alternative methods, NIRS is widely employed due to its capability to rapidly and accurately determine target quality components [21]. Therefore, the establishment of a NIRS prediction model can offer a more efficient and convenient approach for quality prediction and adaptation selection in diverse regions of common vetch. The accuracy and reliability of NIRS are widely believed to be primarily determined by the number of samples modeled and the variability of components [22]. To establish a more precise calibration model for quantitative analysis of nutrients and mineral elements in common vetch, we utilized the average spectrum from 190 different varieties of common vetch for modeling. The findings from this study demonstrate diversity and coefficient of variation in the nutritional quality among the selected samples, which can be attributed to variations in common vetch varieties. Therefore, it is essential to incorporate certain levels of variability in sample nutritional quality not only for establishing an accurate and comprehensive NIRS model but also ensuring its applicability for future predictions across various sample types and component contents [23].

In this study, we discovered that the original spectrum and spectral pattern of common vetch were consistent with previous research findings [24]. The spectrum of common vetch exhibits five prominent absorption peaks across the entire wavelength range, specifically located at approximately 1,450, 1,740, 1,910, 2,120 and 2,350 nm. Previous studies have indicated that within the ranges of 1,150 to 1,250, 1,400 to 1,650, 2,050 to 2,150 nm, as well as at wavelengths of 2,250 and 2,350 nm, C-H and O-H overtones originating from carbohydrates and sugars play a dominant role [25]. Cellulose C-H and O-H bonds exhibit signals at wavelengths around 1,470, 1,780, 1,845 and 2,085 nm. N-H overtone absorption bands are associated with proteins; these associations can be observed at approximately 1,455, 1,555, 1,742.5, 2,087.5 and 2,182.5 nm [26]. The peak value for the C-O stretchy overtone signal is close to 2,303.75 nm and the peak value for O-H bond sin water molecules appear sat approximately 1,432.5 and 1,932.5 nm [27].

To enhance the accuracy and reliability of the NIRS calibration model, most studies typically partition the sample subset into a calibration set and a validation set [28]. For this study, the calibration and validation sets were randomly allocated at a ratio of 4:1 based on modified partial least squares score. PCA analysis revealed that both validated and calibrated samples exhibited similar distributions and comparable ranges in terms of reference values, indicating their suitability for establishing and verifying NIRS equations. Subsequently, employing MPLS regression technology along with different wavelength ranges, mathematical processing techniques, and scattering correction methods resulted in the generation of 30 equations for each parameter index. The optimal model is selected by combining R^2^_C_ and R^2^_CV_.

R^2^_C_ values indicate the extent to which the model fits the calibration set. In NIRS analysis, a high R^2^_C_ value indicates that the model can effectively elucidate the relationship between the absorption peaks of the calibration spectra and the properties of the samples, which enables us to identify the most stable and reliable models [29]. In this study, we observed that the R^2^_C_ effect of K, P, Mg, and Fe is relatively limited compared to other indicators. This could be attributed to the narrow concentration range of these elements and their insufficient combination with organic compounds such as amino acids, proteins, and carbohydrates in the sample [30]. Additionally, a significant portion of these elements exist in the form of free ions within the sample without any absorption effect in the near-infrared spectral region. Consequently, no absorption peak is generated which hampers the establishment of a near-infrared prediction model [31]. In addition, modeling elements may need to consider a variety of chemical forms and states (such as organic carbon versus inorganic carbon), and these complex reactions and states may lead to challenges in the accuracy of the model, resulting in K, P, Mg, and Fe modeling effects greatly affected by the complexity of the sample [32]. In this study, the R^2^_C_ values for DM, CP, NDF, ADF, ash, EE, and OM all exceeded 0.90. This high correlation can be attributed to the derivative and scattering correction treatment of the spectrum, which establishes a precise relationship between the spectral data and the nutrient content of common vetch at specific wavelengths and absorption intensities [33]. Studies have demonstrated that the integration of derivative analysis with descattering treatment markedly enhances the R^2^_C_ value in establishing the predicted nutrient composition of beans, a finding that aligns with the results of this study [34]. In NIRS, the R^2^_CV_ primarily serves to assess the model’s consistency and accuracy concerning spectral features and sample properties across various data subsets derived from the calibration set [35]. In this study, the R^2^_CV_ values for DM, CP, NDF, ADF, ash, EE, and OM all exceeded 0.90. This high performance can be attributed to the selection of common vetch samples from diverse varieties and regions, ensuring a representative and varied dataset that enhances the model’s generalization capability [36]. Additionally, appropriate preprocessing methods were employed during modeling, and an adequate amount of data was utilized, which further contributed to the elevated R^2^_CV_ values. The low R^2^_CV_ values observed for P, Mg, K, and Fe in this study may be attributed to the relatively weak absorption signals of complex compounds formed by these mineral elements in the near-infrared spectrum. These weak signals are susceptible to interference and noise from other components, thereby complicating the model’s ability to accurately establish a quantitative relationship between these elements and the spectral data [37].

The validation set was utilized to assess the predictive capability of the NIRS model for determining nutrient and mineral element content in external common vetch samples. By validating the prediction set model, we observed that the RMSEP values for common vetch indicators ranged from 0.01 to 1.87, with CP, NDF, ADF, and ash exhibiting RMSEP values exceeding 1.0 while other indicators demonstrated values below 1.0. R^2^_p_ values for P, K, and Mg were found to be 0.78, 0.80, and 0.70 respectively indicating relatively lower prediction accuracy, whereas R^2^_p_ values for other nutrients and mineral elements ranged from 0.88 to 0.96. R^2^_p_ is a critical metric for evaluating the accuracy of model predictions on independent test sets. In this study, the R^2^_p_ values for DM, CP, NDF, ADF, ash, and OM were consistently high. Carbas et al demonstrated that R^2^_p_ values are positively correlated with R^2^_c_ and R^2^_cv_ when using near-infrared spectroscopy to assess nutritional and anti-nutritional parameters in common legumes, findings which are consistent with our results [38]. Furthermore, the RMSEP value obtained in this study is relatively low, signifying minimal prediction error and superior model accuracy, which contributes significantly to the elevated R^2^_p_ value observed [39].

In terms of RPD value, the values of P, K, and Mg are all below 2.5, whereas the RPD values of other indicators exceed 2.5. Based on a threshold of RPD≥2.5, the prediction results obtained from the constructed model demonstrate accuracy and applicability in practical quantitative analysis. When 2.0<RPD≤2.5, it indicates relatively lower prediction accuracy of the constructed model and is suitable for preliminary screening purposes only. If RPD<2.0, it implies that the effectiveness of the constructed model is significantly reduced and cannot be applied in actual production practice [40]. In this study, the prediction model for P, K, and Mg exhibited relatively poor performance, possibly due to the limited variability in their contents, weak correlation with certain major elements in the sample, inadequate sample size and range of variation, as well as alterations in their chemical bonds caused by fluctuations in temperature and humidity within the external environment during sample storage [41]. It is plausible that the absorption characteristics of P, K, and Mg in the near-infrared spectral region are either weak or significantly overlap with the spectral signatures of other components [32]. This complicates the accurate extraction of characteristic information for these elements from the spectrum, thereby impacting the prediction accuracy of the model and leading to a lower RPD value [42]. In this study, the RPD value for CP exceeds 4.0, indicating a high level of prediction accuracy [18]. The protein is abundant in N-H groups, which exhibit distinct characteristic absorption peaks in the near-infrared (NIR) spectral region. The position, intensity, and shape of these absorption peaks are closely associated with the chemical environment of the N-H groups and demonstrate a strong linear correlation with protein content [43]. By effectively capturing these features, the model establishes a robust correlation between spectral information and protein content, thereby minimizing the deviation between predicted and actual values and significantly enhancing the RPD value. In this study, the RPD values of NDF, ADF and OM all exceeded 3. This phenomenon can be attributed to the large number of C-H and O-H functional groups in these nutrients, which enable the model to effectively capture their characteristics and accurately correlate the spectral characteristics with the nutrients, thereby reducing the deviation between the predicted value and the actual value and thereby increasing the RPD value [44]. Furthermore, the modeling samples encompass a broad spectrum of sources and conditions, enabling the model to capture the spectral characteristics and content relationships of NDF, ADF, and OM across diverse scenarios [45]. For instance, when modeling common vetches, the samples include various common vetches grown under different environmental conditions, thereby enhancing the model’s adaptability to variations in NDF, ADF, and OM contents and improving prediction accuracy, which in turn increases the RPD value. In contrast, the RPD value for ash is relatively low, primarily because crude ash does not occur in isolation within actual samples. The surrounding organic components, such as proteins and carbohydrates, interfere with the weak spectral signals of mineral elements in ash [46]. This interference makes it challenging for the model to extract relevant spectral features associated with ash, thereby affecting the accurate prediction of ash content and consequently reducing the RPD value [47].

The accuracy of the model for P, K, and Mg contents is sufficient for screening purposes, while other parameter indicators can accurately predict their values and be applied in actual production. Some studies have evaluated the modeling efficacy of mineral element content in common raw beans. The findings indicate that the predictive performance for certain mineral elements is suboptimal, allowing only for preliminary screening [48]. This discrepancy may be attributed to interactions between mineral elements and nutrients components, leading to overlapping absorption peaks of different nutrients in the near-infrared spectral region [49]. Additionally, the formation of complexes between some mineral elements (such as Mg) and organic acids alters the original spectral characteristics of these elements, causing them to deviate from the typical absorption patterns observed in near-infrared spectra. Consequently, the overall prediction accuracy of the model is diminished [50]. The difference in results shown in this study may also be caused by the above results. Numerous studies have demonstrated the immense potential of NIR in screening quality traits of diverse plants [51,52]. This approach will also facilitate future cultivation of different varieties of common vetch. In forthcoming research, near-infrared spectroscopy could be employed to forecast genetic data of common vetch for enhanced screening of high-quality varieties.

The NIR model established in this study can be utilized for the screening of high-quality varieties of common vetches, including those with elevated protein content and fiber content, to facilitate the cultivation of mixed forage. This investigation not only presents a rapid, convenient, cost-effective, and accurate screening method for the efficient application of common vetch but also offers a robust strategy for its future large-scale cultivation and utilization.

## CONCLUSION

In this study, NRIS coupled with a MPLS method was employed to develop a rapid and reliable analytical technique for determining nutrients and elements in common vetch. The results demonstrated that for DM, CP, NDF, ADF, ash, and OM, the R^2^_p_ exceeded 0.90, and the RPD surpassed 2.5. For Ca, P, K, and Fe, R^2^_p_ values ranged from 0.70 to 0.91, and RPD values exceeded 2.0. These findings suggest that the proposed model offers an efficient and accurate means for evaluating the quality of common vetch. This research holds significant theoretical and practical implications for enhancing the broad and efficient utilization of common vetch resources.

## Figures and Tables

**Table 1:** Spectral line, standard curve, and correlation coefficient of each element

Element	Analytical line (nm)	Standard curve equation	Correlation coefficient
Ca	211.276	Y^1)^ = 105.3169x^2)^+40.2397	0.99963
K	766.491	Y = 293.3144x+504.6263	0.9999
P	253.561	Y = 315.9378x+19.7925	0.99984
Mg	202.582	Y = 198.1616x+13.0559	0.99976
Fe	238.204	Y = 6,202.9591x+18.7043	0.99975

1) Y, spectral absorption intensity.

2) x, elemental concentration.

**Table 2:** Basic statistical data on the nutrient content and elemental composition of common vetch (DM basis)

Parameter	Min (%)	Max (%)	Mean (%)	SD	CV (%)^1)^
DM	91.08	94.71	93.15	0.68	0.73
CP	7.17	38.21	17.71	5.26	29.70
NDF	29.34	58.55	40.25	5.37	13.34
ADF	19.19	43.57	28.68	4.24	14.78
Ash	3.92	16.53	10.13	2.89	28.53
EE	1.19	3.56	2.40	0.45	18.75
OM	76.14	90.00	83.02	3.19	3.84
Ca	0.93	3.12	1.77	0.47	26.55
P	0.13	0.42	0.25	0.07	28.00
K	0.74	3.93	2.21	0.75	33.94
Fe	0.01	0.16	0.06	0.03	55.93
Mg	0.23	0.51	0.35	0.06	17.14

1) CV = (Standard deviation/Mean)×100.

DM, dry matter; SD, standard deviation; CV, coefficient of variation; CP, crude protein; NDF, neutral detergent fiber; ADF, acid detergent fiber; EE, ether extract; OM, organic matter.

**Table 3:** Establishing and selecting an optimized NIRS model for the assessment of nutrient composition and elemental content in common vetch

Component	Sample number	Treatment	SD	REMSEC	R^2^C	REMSECV	R^2^CV
DM	152	Scale and linear (1,4,4,1)	0.66	0.18	0.93	0.21	0.90
CP	152	Weighted MSC (1,4,4,1)	5.05	0.97	0.96	1.12	0.96
NDF	152	Inverse MSC (0,0,1,1)	5.17	1.25	0.94	1.37	0.93
ADF	152	SNV and detrend (1,4,4,1)	4.14	0.93	0.95	1.04	0.94
Ash	152	Scale and quadratic (1,4,4,1)	2.83	0.47	0.97	0.59	0.96
EE	152	SNV and detrend (1,4,4,1)	0.43	0.10	0.95	0.14	0.90
OM	152	Weighted MSC (1,4,4,1)	3.13	0.43	0.98	0.56	0.97
Ca	116	Detrend only (1,4,4,1)	0.47	0.11	0.95	0.13	0.92
P	152	Detrend only (1,4,4,1)	0.06	0.02	0.89	0.03	0.82
K	116	SNV only (1,4,4,1)	0.77	0.22	0.92	0.26	0.86
Mg	116	Weighted MSC (0,0,1,1)	0.17	0.02	0.88	0.03	0.82
Fe	116	Scale and quadratic (1,4,4,1)	0.03	0.01	0.79	0.01	0.74

NIRS, near-infrared reflectance spectroscopy; SD, standard deviation; REMSEC, root mean standard error of calibration; R^2^C, coefficient of determination of calibration; REMSECV, mean standard error of cross-validation; R^2^CV, coefficient of determination of validation; DM, dry matter; CP, crude protein; NDF, neutral detergent fiber; ADF, acid detergent fiber; EE, ether extract; OM, organic matter.
